# Topological insulators for thermoelectrics: A perspective from beneath the surface

**DOI:** 10.1016/j.xinn.2024.100782

**Published:** 2025-01-22

**Authors:** Michael Y. Toriyama, G. Jeffrey Snyder

**Affiliations:** 1 Northwestern University, Evanston, IL 60208, USA

**Keywords:** thermoelectrics, topology, band inversion, band warping

## Abstract

Thermoelectric properties of topological insulators have traditionally been examined in the context of their metallic surface states. However, recent studies have begun to unveil intriguing thermoelectric effects emerging from the bulk electronic states of topological insulators, which have largely been overlooked in the past. Charge transport phenomena through the bulk are especially important under typical operating conditions of thermoelectric devices, necessitating a comprehensive review of both surface and bulk transport in topological insulators. Here, we review thermoelectric properties that are uniquely observed in topological insulators, placing special emphasis on unconventional phenomena emerging from bulk states. We demonstrate that unusual thermoelectric effects arising from bulk states, such as band inversion-driven warping, can be discerned in experiments through a simple analysis of the weighted mobility. We believe that there is still plenty to uncover within the bulk of topological insulators, yet our current understanding can already inspire new strategies for designing and discovering new materials for next-generation thermoelectrics.

## Public summary

• Thermoelectric devices offer broad applications, such as eco-friendly cooling.
• Topological materials are emerging as key players in high-performing thermoelectrics.
• Topological insulators, which are known for their unique surfaces, possess exceptional bulk properties.
• A review of bulk thermoelectric effects arising from e.g. band inversion-driven warping is given.
• Strategies for analyzing and engineering topological insulators for thermoelectric applications are reviewed.

## Graphical abstract

**Figure:**
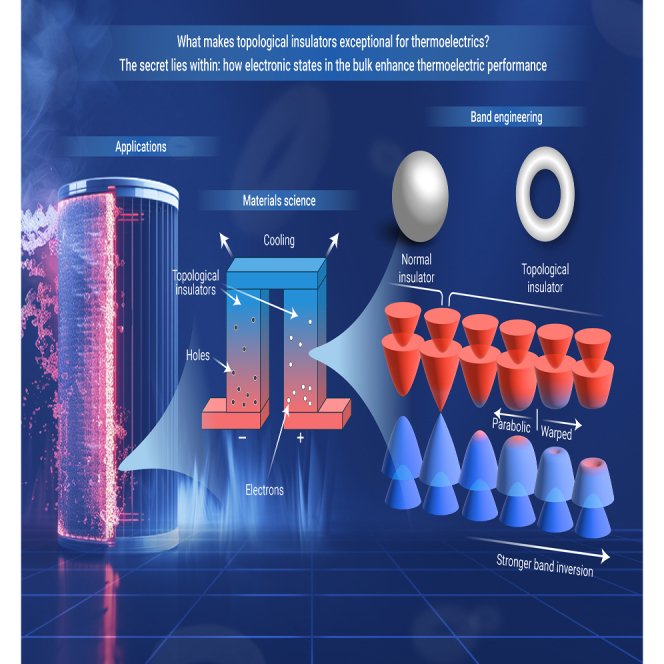

## Introduction

Thermoelectric (TE) materials possess the unique ability to convert thermal energy to electrical energy, and vice versa, allowing niche applications in energy conversion technologies across various domains. In generation mode (i.e., heat to electricity), TEs can be used to power remote devices such as deep-space probes^1^ and Internet of Things sensors,^2^ as well as to recover energy from waste heat in industrial settings.^3^^,^^4^ Conversely, in Peltier cooling mode (i.e., electricity to heat pumping), TE devices are used for targeted temperature control of optoelectronic and photonic devices^5^^,^^6^ and serve as eco-friendly alternatives to traditional cooling systems that rely on vapor compression technologies.^7^^,^^8^ The wide-ranging applications of TEs can potentially address (1) the ever-growing societal demand for energy, particularly from cooling,^9^ and (2) the global target of net-zero emissions by 2050. Achieving high TE power conversion efficiency is therefore critical for realizing such potential.

The efficiency of a TE device is critically dependent on the material used. The performance is determined by the material figure of merit $zT=S^{2}\sigma T/\kappa$, where S is the Seebeck coefficient, σ is the electrical conductivity, and κ is the thermal conductivity. These material properties are interconnected and often conflict with one another (e.g., increasing σ can also increase κ), making it challenging to optimize zT in a material.^10^ Material engineering and even material discovery are, therefore, key areas for pushing the boundaries of TE technologies.

In the long history of TE research, numerous classes of materials have been examined. Topological insulators (TIs) are a relatively new class of materials that can be characterized as metal-like on the surface and semiconductor-like in the bulk.^11^ In terms of the electronic structure, TIs possess gapless states at the surface while simultaneously having a band gap in the bulk, making them promising for applications such as low-temperature spintronics.^12^ At the same time, some TIs, notably ${\text{Bi}}_{2}{\text{Te}}_{3}$-based materials, happen to be some of the best TE materials today and are used in commercially available TE devices. Because of this, a complete understanding of the transport properties in TIs is of practical importance for current, and future, TE technologies.

**Figure 1:**
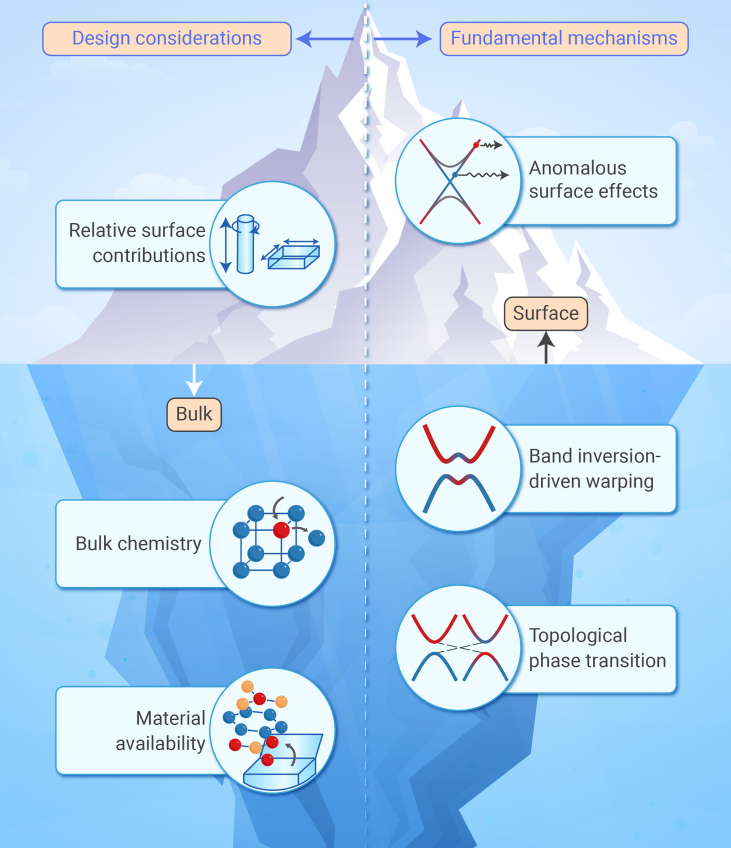
The “knowledge iceberg” of topological insulators in the context of thermoelectric properties and design While surface states and emergent surface properties of topological insulators are well known, there is much to uncover beneath the surface, i.e., unique thermoelectric effects arising from bulk electronic states.

With the recent surge in quantum materials research, the community has developed a strong fundamental understanding of atypical TE effects in TIs, particularly those arising from surface states such as geometric size dependencies and the anomalous Seebeck effect. However, comparatively less attention has been dedicated to transport phenomena in the *bulk* of TIs. Recent studies have begun to show that there are unconventional TE effects that arise from bulk electronic states in TIs, such as band inversion-driven warping leading to high valley degeneracy. We therefore view the current state of the cross-disciplinary field as a “knowledge iceberg” (Figure 1); quite literally, there are fascinating phenomena hidden *beneath the surface* of TIs that are yet to be explored.

Unlike other reviews on the topic,^13^^,^^14^^,^^15^^,^^16^^,^^17^ which often discuss TE effects arising from TI surface states (and, nonetheless, serve as valuable milestones in the field), we provide a comprehensive review of how *bulk properties* unique to TIs affect TE transport properties. We begin with a short summary of how TI surface states impact TE properties, drawing from over a decade of experimental and theoretical research. At the core of this work, we review recent developments in understanding the bulk electronic structure and bulk charge transport properties of TIs. We demonstrate that unconventional phenomena stemming from bulk states, such as band inversion-driven warping, can be discerned in experiments by performing a rather simple analysis of the weighted mobility. Although the broader class of topological materials can also exhibit unconventional phenomena such as the anomalous Nernst effect,^16^^,^^18^ our focus here is limited to TIs. We hope that this review encourages further research into the unique properties of TIs, especially those emerging from bulk states.

## Topological surface states and thermoelectric properties

**Figure 2:**
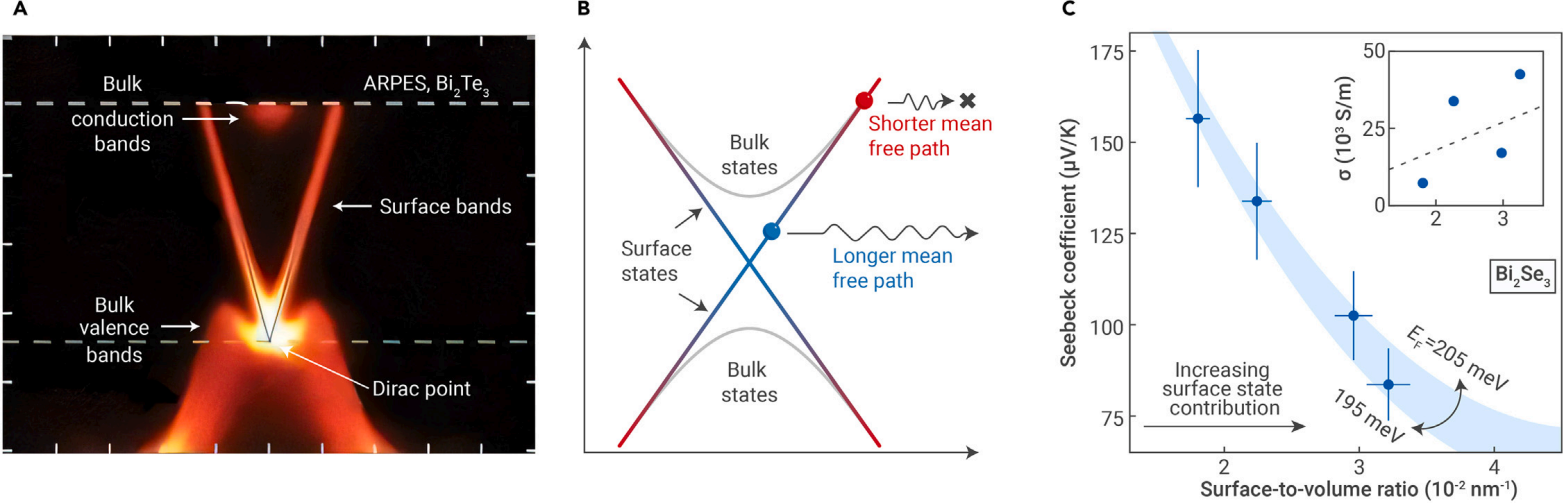
Topological surface states and thermoelectric properties (A) Electronic structure of ${\text{Bi}}_{2}{\text{Te}}_{3}$ from angle-resolved photoemission spectroscopy (ARPES). The bulk valence bands and bulk conduction bands are separated by a band gap, yet the gapless surface bands cross the bulk band gap. Image adapted from Chen et al.^19^ with permission. Copyright 2009, American Association for the Advancement of Science. (B) Schematic of the electronic structure of a topological insulator. Surface carriers in the bulk band gap, which are protected against backscattering by time-reversal symmetry, have a longer mean free path than surface carriers in the bulk bands, which are subjected to bulk-surface interactions. (C) Seebeck coefficient and electrical conductivity (inset) of ${\text{Bi}}_{2}{\text{Se}}_{3}$ nanowires with varying surface-to-volume ratios. Error bars are indicated for the Seebeck coefficient. Within approximately the same Fermi level range (shaded region), the conductivity may increase, but the Seebeck coefficient drops as the surface states, with opposite signs of thermopower, contribute more. Image adapted from Shin et al.^23^ with permission. Copyright 2016, Royal Society of Chemistry.

The prospect of using TIs for TE applications has historically been motivated by the idea of leveraging TI surface states to enhance zT. The surge in TI research in recent years has resulted in advanced techniques for probing the gapless surface states of TIs, such as angle-resolved photoemission spectroscopy (ARPES) as shown for ${\text{Bi}}_{2}{\text{Te}}_{3}$ in Figure 2A.^19^ It has also resulted in a better understanding of exotic phenomena not normally observed in conventional (non-TI) materials, arising from the distinct charge transport behaviors along the surface and through the bulk of a material. For example, the scattering behavior of TI surface states can be strongly energy dependent due to interactions between the bulk and surface states (Figure 2B), which can result in a sign anomaly in the Seebeck coefficient where, e.g., an n-type material exhibits a positive S.^20^^,^^21^ The phenomenon is sometimes called the *anomalous Seebeck effect*, where the bulk and surface carriers have opposite signs for the thermopower. Because the overall (measurable) Seebeck coefficient is a conductivity-weighted average of the surface and bulk thermopowers,^16^ surface states should have a subtractive effect due to their opposite sign,^22^ similar to bipolar conduction effects in bulk intrinsic semiconductors that have both electrons and holes.

Although theoretical predictions often propose that zT in TIs can be enhanced by balancing surface and bulk charge transport,^24^^,^^25^^,^^26^ there are important factors that must be considered to utilize the surface and bulk channels synergistically. (1) Surface area. The surface area of a sample compared to the bulk volume determines the degree to which surface states affect TE properties, making geometric factors and processing conditions important considerations for TIs.^27^ By increasing the surface-to-volume ratio,^23^ which leads to higher relative contributions from surface states, a corresponding decrease in S has been observed in TIs such as ${\text{Sb}}_{2}{\text{Te}}_{3}$^28^ and ${\text{Bi}}_{2}{\text{Se}}_{3}$^23^ (Figure 2C). Typically, the effects of TI surface states are pronounced when the sample dimensions are on the order of a few nanometers. In a sufficiently thin film, surface states on opposite surfaces of a TI can also hybridize and introduce a band gap.^29^^,^^30^ (2) Temperature. Due to electron-phonon scattering,^24^ the influence of surface states may become overwhelmed by bulk states as temperature increases.^31^ Therefore, surface state effects tend to be significant only at temperatures below ∼200 K,^32^^,^^33^ which limits their applicability. (3) Fermi level ($E_{F}$). In general, the $E_{F}$ position in a TE material strongly affects charge transport properties and, as a result, zT. In TIs, the $E_{F}$ position is important because it modulates the relative contributions from bulk and surface states to TE properties.^24^^,^^25^^,^^26^ The $E_{F}$ position is also important when attributing observed changes in TE properties to mechanisms related to TI surface states, because increasing the contributions from surface states has the same effects on TE properties as increasing the carrier concentration: both lead to an increase in σ and a decrease in S. The $E_{F}$ position can be estimated by supplementing experimental measurements with transport modeling^23^^,^^34^ or using magnetotransport measurements.^35^

## Bulk properties in topological insulators for thermoelectrics

TIs have generated widespread interest because of their protected surface states, often overshadowing investigations into their bulk properties. Yet, materials used in TE devices are typically on the scale of millimeters or larger, operate at room temperature or above, and are doped to carrier concentrations on the order of $10^{19}$ ${\text{cm}}^{-3}$ and above, where the effects of surface states are less pronounced and potentially outweighed by bulk transport. Recent findings have revealed that unconventional transport phenomena can also emerge from bulk TI states, driven fundamentally by band inversion. In this section, we redirect our attention to bulk TE effects that are unique to TIs, opening up new opportunities to enhance zT in practical applications.

### Phenomenological bulk effects unique to topological insulators

#### Band inversion-driven warping

**Figure 3:**
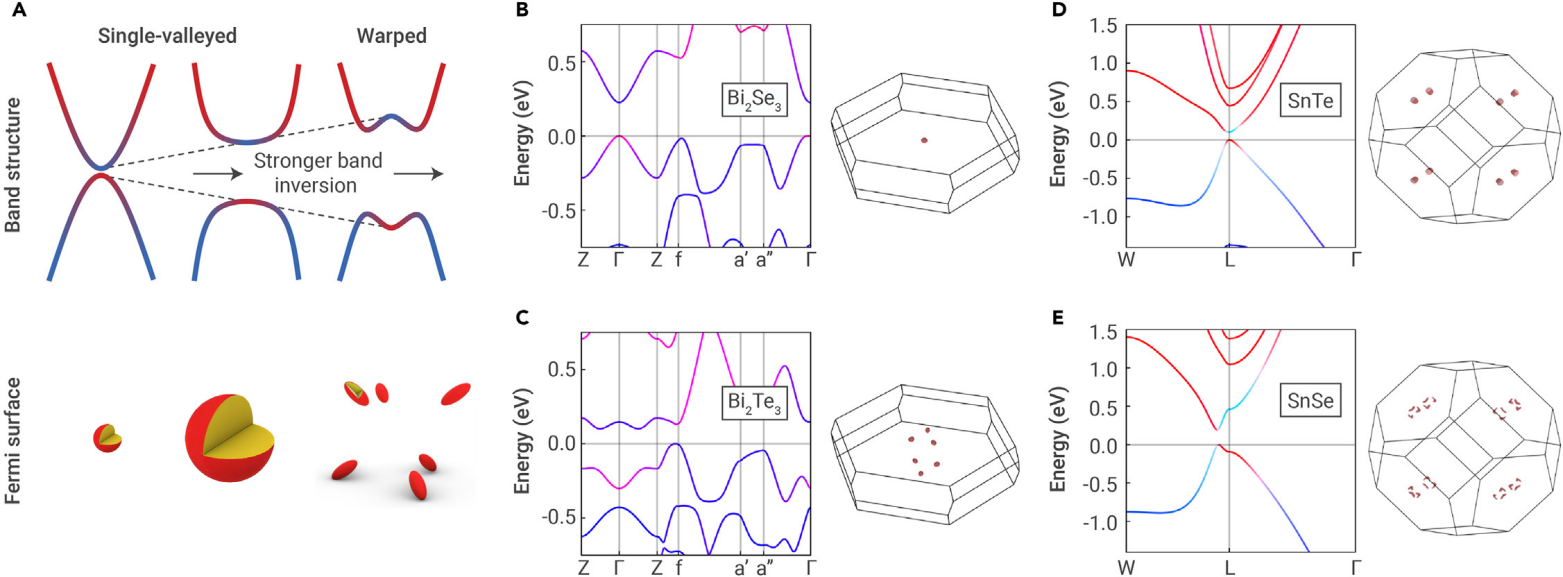
Bulk band structures of topological insulators (A) Illustration of the bulk band structure and corresponding isoenergy Fermi surface near the band edge, and how they evolve with band inversion strength in a topological insulator. With stronger band inversion, the bands become warped, and the Fermi surface becomes multi-valleyed. (B–E) The band structure and Fermi surface calculated using *ab initio* methods are shown for (B) ${\text{Bi}}_{2}{\text{Se}}_{3}$, (C) ${\text{Bi}}_{2}{\text{Te}}_{3}$, (D) SnTe, and (E) rock-salt SnSe.

One of the most important material properties for TEs is the valley degeneracy (i.e., the number of carrier pockets that are contributing to charge transport in the bulk). Higher valley degeneracy improves the power factor, provided that intervalley scattering effects are not significant.^36^^,^^37^ In TIs, the valley degeneracy is fundamentally linked to band inversion.^38^ Assuming band inversion occurs at a k-point labeled $k_{0}$, strong band coupling can offset the band edges from $k_{0}$, leading to the formation of multiple carrier pockets (Figure 3A).^38^ We refer to this phenomenon as *band inversion-driven warping*; the term warping refers to the nonparabolic “W”-like (“M”-like) shape adopted by the conduction (valence) band as opposed to the conventional “U”-like (“upside-down U”-like) shape, as shown in Figure 3A.

Band inversion is necessary for band warping in TIs; however, it is not sufficient. There are several examples of TIs that, despite being chemically similar, possess different band structure shapes (warped vs. non-warped) and, therefore, different valley degeneracies. A notable pairing is ${\text{Bi}}_{2}{\text{Se}}_{3}$ and ${\text{Bi}}_{2}{\text{Te}}_{3}$. While both TIs possess inverted bands at the Γ-point, the bands in ${\text{Bi}}_{2}{\text{Se}}_{3}$ are centered at the Γ-point and therefore not warped, resulting in a valley degeneracy of 1 (Figure 3B). On the other hand, the bands are warped at the Γ-point in ${\text{Bi}}_{2}{\text{Te}}_{3}$, moving the band edges to *k*-points on the bisectrix plane of the Brillouin zone.^39^ This results in a valley degeneracy of 6 (Figure 3C). In alloys of ${\text{Bi}}_{2}{\text{Te}}_{3}$, there is a possibility that multiple warped bands contribute to an even higher valley degeneracy of up to 12,^40^ as suggested by ARPES measurements.^41^ Additionally, in IV–VI rock-salt TIs, band inversion occurs at the L-point.^42^ While the bands in SnTe are centered at the L-point and not warped so that the valley degeneracy is 4 (Figure 3D), bands in the metastable rock-salt phase of SnSe are warped so that the band edges are offset from the L-point, giving rise to a valley degeneracy of 24 (Figure 3E).

Recently, a k·p perturbation theory-based band structure model was used to derive a mathematical condition for when inverted bands become warped in TIs that are centrosymmetric and obey time-reversal symmetry (e.g., non-magnetic TIs).^38^ The model elucidated the importance of the *band inversion strength* of a TI, $M_{0}$, where its magnitude is defined as

(Equation 1) $$\left|M_{0}\right|=\frac{E_{\text{CB}}\left(k_{0}\right)-E_{\text{VB}}\left(k_{0}\right)}{2}\text{,}$$

where $E_{\text{CB}}$ and $E_{\text{VB}}$ are the conduction and valence band edges, respectively, and $k_{0}$ is the k-point where band inversion occurs. In other words, $\left|M_{0}\right|$ is a measure of the energy separation between the lowest unoccupied conduction band and the highest occupied valence band at $k_{0}$. For a TI, the sign of $M_{0}$ is negative, and more negative values correspond to stronger band inversion. Note that normal insulators with non-inverted bands can also be represented using Equation 1 but with a positive $M_{0}$.

The k·p model showed mathematically that, when $M_{0}$ is sufficiently negative and past a certain threshold value, the bands in a TI become warped. Physically, this means that the bands must be sufficiently inverted for warping to occur in a TI (Figure 3A). A more thorough derivation and discussion of the mathematical condition can be found in Toriyama and Snyder.^38^^,^^43^ This band inversion-driven warping phenomenon can explain the distinct band structure shapes between ${\text{Bi}}_{2}{\text{Se}}_{3}$ and ${\text{Bi}}_{2}{\text{Te}}_{3}$. Indeed, the energy separation between the conduction and valence bands at the Γ-point is larger in ${\text{Bi}}_{2}{\text{Te}}_{3}$ ($M_{0}$ = −0.23 eV in Figure 3C) than in ${\text{Bi}}_{2}{\text{Se}}_{3}$ ($M_{0}$ = −0.12 eV in Figure 3B), thereby explaining the warped bands in ${\text{Bi}}_{2}{\text{Te}}_{3}$ and the non-warped bands in ${\text{Bi}}_{2}{\text{Se}}_{3}$. Because the band inversion strength $M_{0}$is a characteristic of the band structure, it is principally modulated by (1) spin-orbit coupling (SOC)^44^^,^^45^^,^^46^ and (2) atomic orbital interactions (e.g., sp-mixing).^47^^,^^48^ Therefore, the stronger band inversion (i.e., more negative $M_{0}$) in ${\text{Bi}}_{2}{\text{Te}}_{3}$, as well as the band warping in ${\text{Bi}}_{2}{\text{Te}}_{3}$, can be attributed to the stronger SOC. Furthermore, the band inversion is stronger at the L-point in cubic SnSe ($M_{0}$ = −0.28 eV in Figure 3E) than in SnTe ($M_{0}$ = −0.05 eV in Figure 3D), providing further credence to the band inversion-driven warping phenomenon. In contrast to the tetradymite compounds, where the stronger SOC in ${\text{Bi}}_{2}{\text{Te}}_{3}$ leads to band warping, sp-mixing determines the band inversion strength in rock-salt compounds.^38^ Atomic orbital interactions can be affected by a number of factors, such as bond lengths and on-site orbital energies.^42^

#### Band warping and thermoelectric performance

Because band inversion-driven warping can lead to the formation of carrier pockets that are close in k-space, intervalley scattering by phonons (especially those near the zone center) may be significant. In general, the benefits of high valley degeneracy on TE properties can be diminished by high scattering rates.^37^ It is, therefore, natural to ask whether the TE performance actually benefits from band warping in TIs.

Shi et al.^44^ initially showed that a complex (warped) band structure in a TI, which can be driven by SOC in materials such as ${\text{Bi}}_{2}{\text{Te}}_{3}$ and ${\text{Bi}}_{2}{\text{Te}}_{2}\text{Se}$, is favorable for the TE power factor. Compared to a parabolic band with a spherical Fermi surface, a complex band structure can increase the area of the Fermi surface at the same volume, therefore enlarging the density of states at a given carrier concentration. Because the density of states effective mass is proportional to the Seebeck coefficient by the Mott relation, a complex band structure can generate a high power factor.

**Figure 4:**
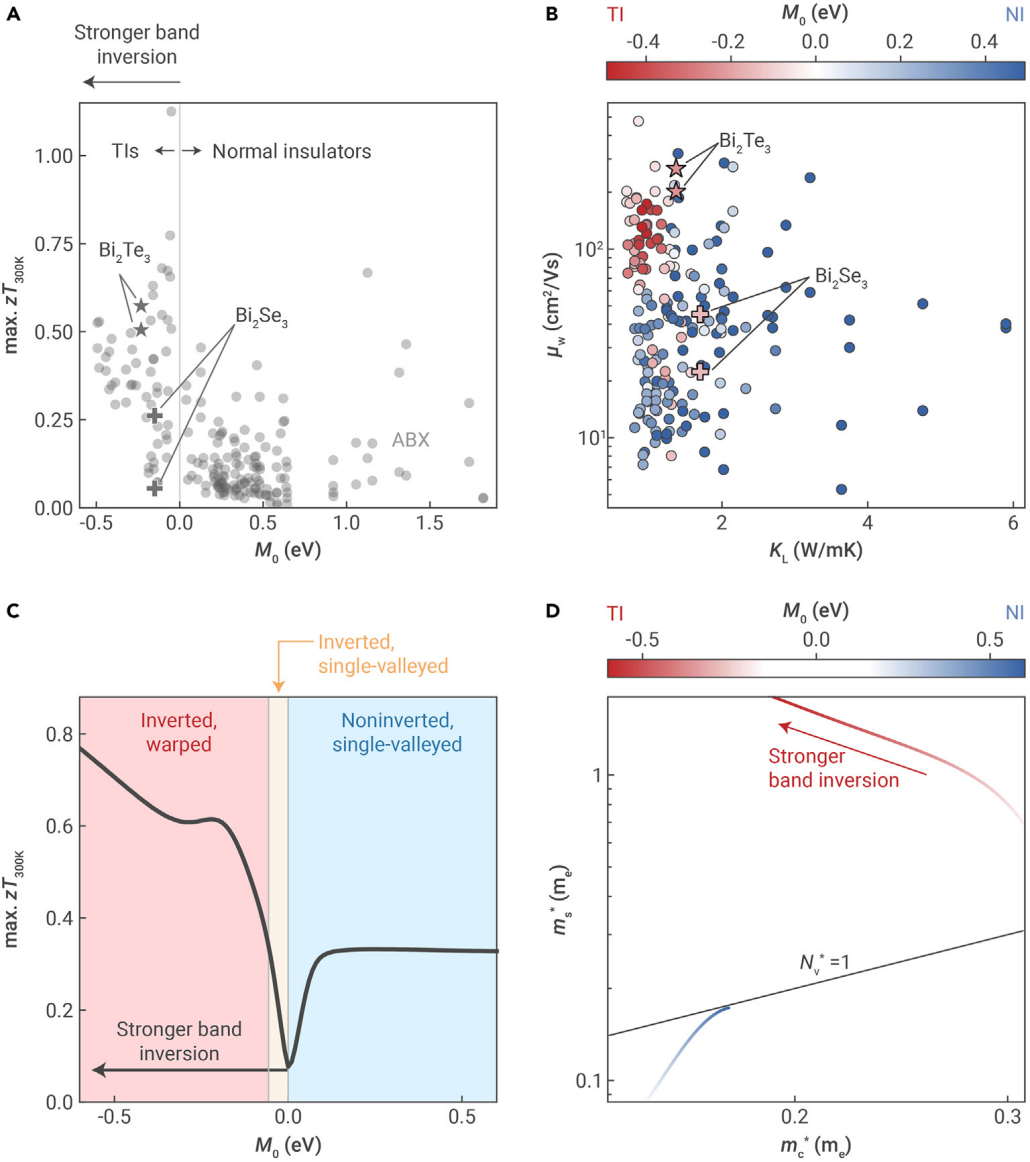
Thermoelectric properties of topological insulators (A) Maximum attainable zT (optimized with respect to doping level) at room temperature, obtained using first-principles Boltzmann transport calculations. The maximum zT is plotted against the $M_{0}$ parameter (defined in Equation 1) which, for topological insulators (TIs), represents the band inversion strength. (B) Weighted mobility $\left(\mu_{w}\right)$ and lattice thermal conductivity $\left(\kappa_{L}\right)$, where TIs are represented by the red coloring (negative $M_{0}$) and normal insulators (NIs) are represented by the blue coloring (positive $M_{0}$). (C) Maximum attainable zT from charge transport modeling using k·p perturbation theory. The background coloring indicates the band structure shape (warped or single valleyed) and topology (inverted or noninverted). (D) Seebeck effective mass $\left(m_{S}^{*}\right)$ and conductivity effective mass $\left(m_{C}^{*}\right)$, where the red and blue coloring represent TIs and NIs, respectively. Images adapted from Toriyama and Snyder^43^ with permission. Copyright 2024, Royal Society of Chemistry.

More recently, the effects of band inversion-driven warping on TE performance were studied by considering a large set of materials using first-principles Boltzmann transport calculations.^43^ It was found that the maximum attainable zT (i.e., the zT value at the optimum doping level) tends to be higher for a TI than a conventional semiconductor and even higher for TIs exhibiting stronger band inversion (Figure 4A). In general, the maximum attainable zT of a material correlates with the TE quality factor $B\propto \mu_{w}/\kappa_{L}$, where higher maximum zT can be explained by higher B.^49^ Here, $\kappa_{L}$ is the lattice thermal conductivity, and $\mu_{w}$ is the weighted mobility. The latter can be interpreted as the carrier mobility (μ) weighted by the density of states,^50^ i.e.,

(Equation 2) $$\mu_{w}=\mu {\left(\frac{m_{S}^{*}}{m_{e}}\right)}^{3/2}=\frac{e\tau }{m_{C}^{*}}{\left(\frac{m_{S}^{*}}{m_{e}}\right)}^{3/2}$$

where τ is the carrier scattering time, $m_{C}^{*}$ is the conductivity effective mass, and $m_{S}^{*}$ is the Seebeck effective mass (which is a measure of the density of states). Further analysis showed that the high TE performance of TIs originates from a combination of low $\kappa_{L}$ and high $\mu_{w}$ (Figure 4B). It is expected that TIs have low $\kappa_{L}$ because band inversion is typically induced by strong SOC interactions, which arises from the presence of heavy atoms. While the high-throughput calculations certainly confirm this, they also reveal that high $\mu_{w}$ is another key advantage of TIs over conventional semiconductors.

The results of first-principles calculations were consistent with charge transport models based on k·p perturbation theory and Boltzmann transport theory.^43^ Namely, the model showed that TIs exhibit high maximum zT that increases nearly monotonically with stronger band inversion, i.e., more negative $M_{0}$ (Figure 4C). The model also showed that the band inversion strength is a critical, if not the most important, material property that determines the maximum zT of a TI. As a result, band inversion-driven warping is the key advantage that TIs uniquely possess over conventional semiconductors for TE applications. Fundamentally, band warping in a TI gives rise to high $\mu_{w}$ (as revealed by first-principles calculations in Figure 4B) by reducing $m_{C}^{*}$ and increasing $m_{S}^{*}$ (Figure 4D; see Equation 2 also).

It is worth emphasizing that stronger band inversion improves the *maximum* zT of a TI, and the carrier concentration must still be optimized to achieve the maximum TE performance. It is therefore more precise to say that strengthening band inversion enhances the TE quality factor B rather than the actual zT. Care must therefore be taken when attributing changes in TE properties to band inversion-related effects, especially when changes in the chemistry are involved (e.g., doping and alloying). In such cases, both the band structure and the Fermi level are likely affected.

### Strategies to improve bulk charge transport properties

Band inversion-driven warping can improve bulk TE properties in TIs and enhance the maximum attainable zT. Therefore, the TE performance can be improved directly by strengthening band inversion (i.e., the degree to which the bands are inverted). The band inversion strength is determined principally by atomic orbital interactions and SOC interactions,^38^ so strategies to manipulate band inversion in a TI should fundamentally aim to modulate these interaction strengths. We discuss some approaches in the following subsections.

It is important to check whether band inversion-driven warping is responsible for affecting TE properties in experiments. This can be directly understood by probing the band structure, using techniques such as ARPES (as in Figure 2A),^19^ the Shubnikov-de Haas effect,^51^^,^^52^ or the de Haas-van Alphen effect.^53^ A more straightforward method is to check the weighted mobility ($\mu_{w}$) given in Equation 2, which can be calculated directly from the measured electrical conductivity and Seebeck coefficient.^50^ $\mu_{w}$ is a robust indicator of warping effects in TIs because it generally increases with the band inversion strength at a given temperature,^43^ although the level of improvement in $\mu_{w}$ will depend on specific features of the material such as orbital chemistry and spin-orbit interactions. The power factor can also be used to understand the electronic properties of a TE material, but it generally depends on the carrier concentration. Accordingly, $\mu_{w}$, which is largely independent of the carrier concentration,^50^ is a better indicator of electronic properties and provides a metric to compare different materials or different states of the same material. As we show in the following sections, analyzing $\mu_{w}$ allows us to provide compelling evidence that band inversion-driven effects are occurring in TIs.

#### Mechanical strain and external pressure

**Figure 5:**
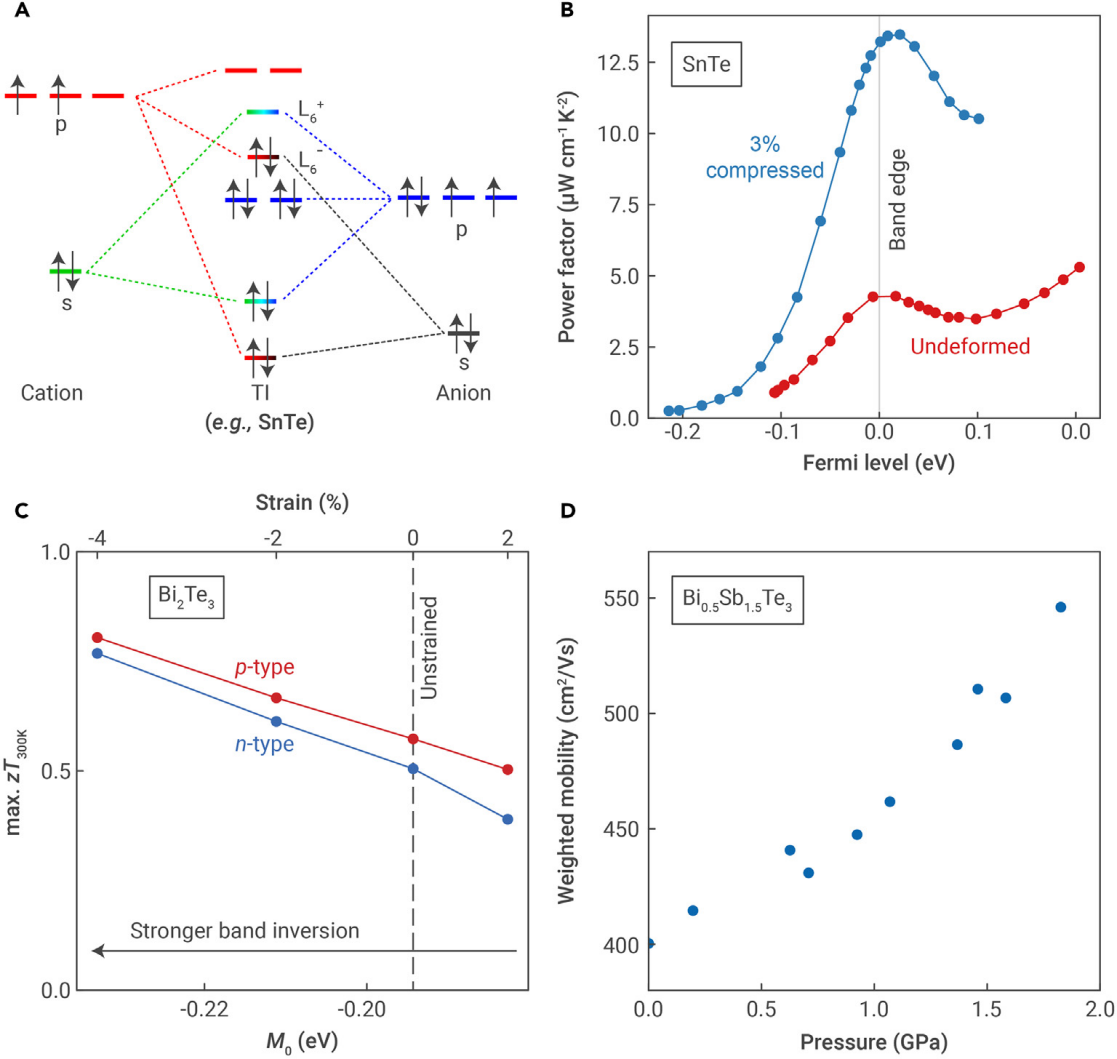
Effects of strain and pressure on thermoelectric properties of topological insulators (A) Molecular orbital diagram for the L-point in IV–VI rock-salt phases, illustrating the inversion of the $L_{6}^{-}$ and $L_{6}^{+}$ states. Image adapted from Toriyama et al.^42^ with permission. Copyright 2022, Royal Society of Chemistry. (B) Calculated n-type power factor of undeformed and compressed SnTe. Data adapted from Dai et al.^57^ (C) Calculated maximum attainable zT of ${\text{Bi}}_{2}{\text{Te}}_{3}$ at different strains, corresponding to varying degrees of band inversion. Image adapted from Toriyama and Snyder^43^ with permission. Copyright 2024, Royal Society of Chemistry. (D) Measured weighted mobility of ${\text{Bi}}_{0.5}{\text{Sb}}_{1.5}{\text{Te}}_{3}$ at different pressures. Data adapted from Bai et al.^58^

In general, applying external pressure or mechanical stress can modify the band structure of a material.^54^^,^^55^ In TIs, strain can affect the band inversion strength by modulating nearest-neighbor orbital interactions.^56^ In rock-salt IV–VI compounds, the energies of the $L_{6}^{-}$ and $L_{6}^{+}$ bands (i.e., those that are involved in band inversion; see Figure 5A) are influenced by sp-mixing between neighboring cations and anions.^42^ From a molecular orbital theory standpoint, compression would enhance mixing between the cation-s and anion-p orbitals, which would raise the $L_{6}^{+}$ band relative to the $L_{6}^{-}$ band and lead to stronger band inversion. First-principles calculations have shown that compressing SnTe leads to warping in the conduction band, splitting the conduction band edge into multiple valleys.^57^ The n-type power factor was correspondingly 3-fold larger than in the non-compressed state for a wide range of Fermi levels (Figure 5B), despite the scattering rates being nearly an order of magnitude higher.^57^

A similar behavior is observed in ${\text{Bi}}_{2}{\text{Te}}_{3}$-like TIs. First-principles calculations have shown that hydrostatically compressing ${\text{Bi}}_{2}{\text{Te}}_{3}$ improves the maximum zT at room temperature for both n- and p-types (Figure 5C). This can be explained by band inversion strengthening, where the $M_{0}$ parameter (defined in Equation 1) becomes more negative with compressive strain in ${\text{Bi}}_{2}{\text{Te}}_{3}$. The $M_{0}$ parameter is determined by the energies of the Bi-$p_{z}$ and Te-$p_{z}$ states at the Γ-point, which make up the valence and conduction band edges, respectively.^59^ Compression enhances both the cation-p/anion-p hybridization and crystal field splitting, which lowers the Bi-$p_{z}$ state relative to the Te-$p_{z}$ state and makes $M_{0}$ more negative.

Strain can be applied through external pressure, and improvements in the power factor with pressure have also been demonstrated experimentally.^58^^,^^60^^,^^61^ From recent measurements of the TI ${\text{Bi}}_{0.5}{\text{Sb}}_{1.5}{\text{Te}}_{3}$,^58^ we find that $\mu_{w}$, which can be calculated from the conductivity and Seebeck coefficient,^50^ increases with applied pressure (Figure 5D). This can be explained as a band inversion-driven effect in a manner similar to ${\text{Bi}}_{2}{\text{Te}}_{3}$.

#### Alloying

Most TE materials are doped and/or alloyed to optimize zT. Since both techniques can affect the band structure and, as a result, the band inversion strength of a TI, transport properties can be regulated in unique ways by altering bulk chemistry. It has been suggested that even trace amounts of doping can affect TE properties, especially when the band structure is sensitive to small changes in SOC interactions.^44^^,^^45^ However, phenomena resulting from band inversion are more readily observed in alloys than doped samples.

**Figure 6:**
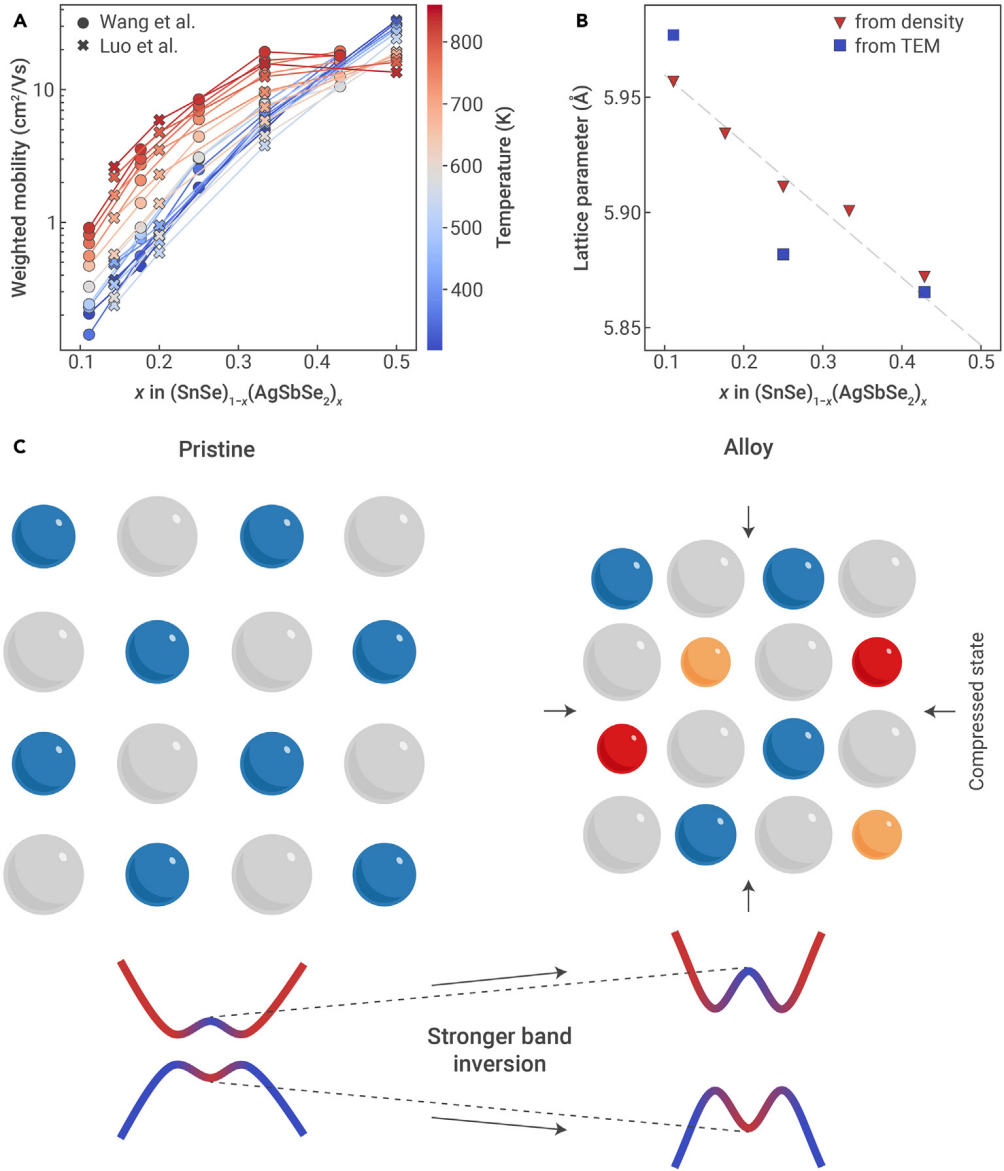
Alloying topological insulators for thermoelectrics (A and B) Weighted mobility (A) and lattice constant (B) of SnSe alloyed with ${\text{AgSbSe}}_{2}$. The weighted mobility is calculated from the reported conductivity and Seebeck coefficient.^50^ Data adapted from Wang et al.^67^ and Luo et al.^68^ (C) Schematic of how alloying can affect the band inversion strength. Alloying-induced compression (i.e., chemical pressure) leads to stronger band inversion in the parent topological insulator phase.

In particular, cubic alloys involving SnSe have shown unconventional TE effects. The metastable rock-salt phase of SnSe is a known TI^62^^,^^63^ with a highly warped band structure (Figure 3E),^42^ and both theory^64^^,^^65^ and experiments^66^ have suggested capitalizing on its unique properties for TEs. Alloying SnSe with ${\text{AgSbSe}}_{2}$, for example, has been shown to improve the power factor across a wide range of temperatures.^67^^,^^68^ A closer examination of the transport properties reveal that $\mu_{w}$ also increases with x in ${\left(\text{SnSe}\right)\;}_{1-x}{\left({\text{AgSbSe}}_{2}\right)\;}_{x}$ (Figure 6A). Normally, $\mu_{w}$ of a two-phase mixture is lower than that of the end members because the carrier mobility is lower. Wang et al. attributed the enhancement of $\mu_{w}$ to the compressive strain in rock-salt SnSe induced by ${\text{AgSbSe}}_{2}$ alloying, which is evidenced by the monotonic decrease in the lattice parameter determined from density and transmission electron microscopy (TEM) measurements (Figure 6B).^67^ Compressing rock-salt SnSe, which is a TI, strengthens sp-mixing and the band inversion strength in a manner similar to compressing SnTe. Since rock-salt SnSe already has a warped band structure (Figure 3E), compression should further warp the band structure (Figure 6C), thus leading to higher $\mu_{w}$. Presently, the increase in $\mu_{w}$ and power factor with the addition of ${\text{AgSbSe}}_{2}$ to SnSe has been substantiated on the basis of lattice compression and band inversion-related effects.^67^ While the justification is certainly reasonable and self-consistent, elemental substitution can also significantly modify the band inversion strength by modulating on-site energies.

Band inversion-related effects have been proposed in other alloy systems as well. Lattice compression and band gap widening were observed in ${\left(\text{PbSe}\right)}_{1-x}{\left(ABX_{2}\right)}_{x}$^69^^,^^70^^,^^71^ similar to cubic alloys of SnSe, which can be explained as a gradual separation of the $L_{6}^{+}$ and $L_{6}^{-}$ bands due to stronger sp-mixing. First-principles calculations of an ordered supercell of ${\left(\text{PbSe}\right)}_{1-x}{\left({\text{AgSbS}}_{2}\right)}_{x}$ ensured that the alloy undergoes a band inversion strengthening-type phenomenon primarily due to lattice compression, despite the chemistry also being affected by introducing Ag, Sb, and S.^71^ Since pristine PbSe is a normal insulator, the observed band inversion-like effects indicate that PbSe likely undergoes a topological phase transition to a TI state upon alloying. In SnTe, the zT was boosted to nearly 1.4 after heavily alloying with GeTe and PbTe and doping with Cd.^72^ Because first-principles calculations revealed that SnTe, upon alloying with GeTe and PbTe, adopts a multi-valleyed band structure, the improvement was attributed in large part to a band inversion-driven phenomenon.^72^

### Topological phase transition

**Figure 7:**
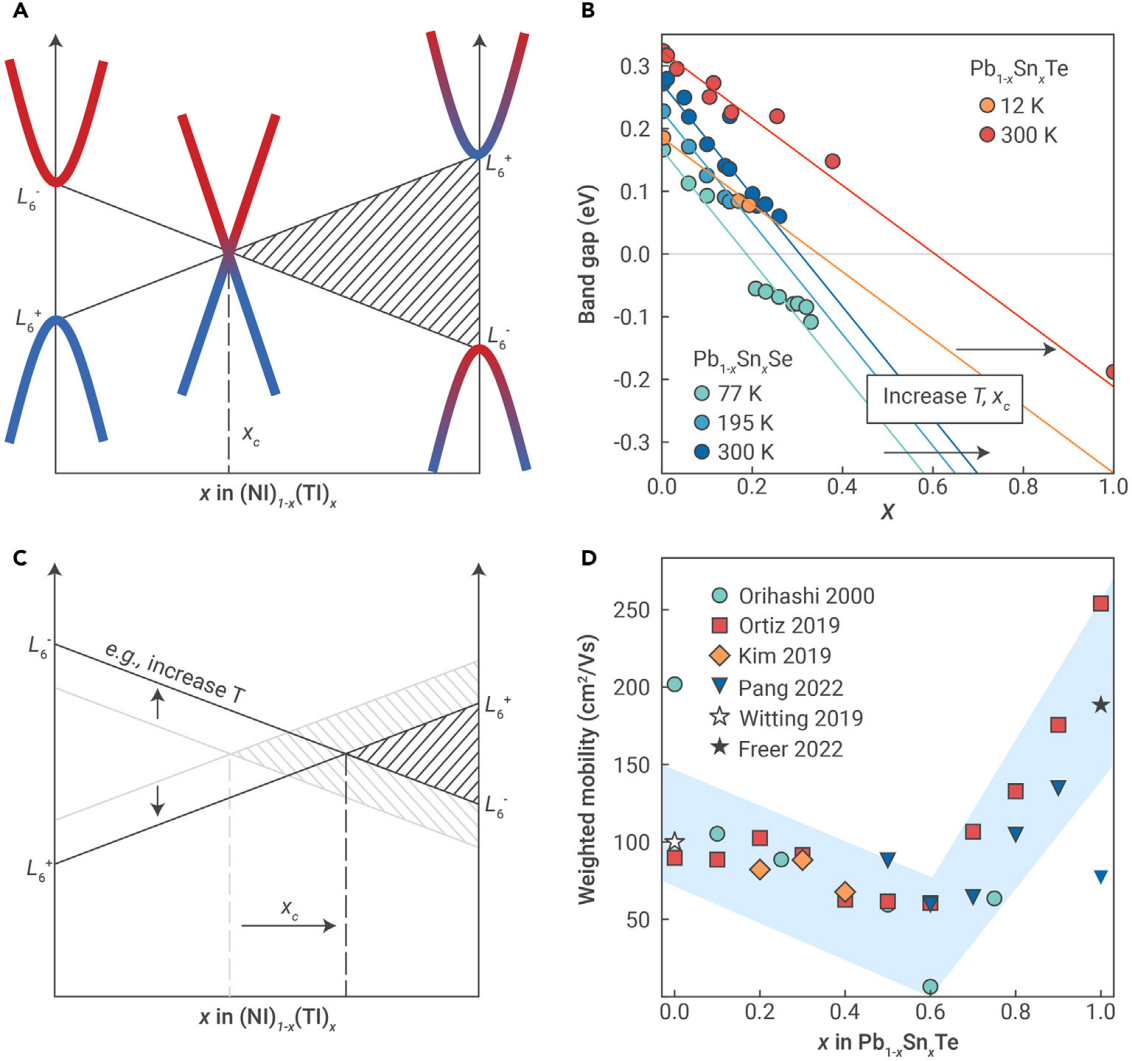
Topological phase transition and thermoelectric properties (A) Schematic of a topological transition in an alloy between a normal insulator (NI) and a topological insulator (TI). In alloys of IV–VI compounds, the transition occurs between the $L_{6}^{+}$ and $L_{6}^{-}$ bands, where the band gap closes at composition $x_{c}$. (B) Band gap of ${\text{Pb}}_{1-x}{\text{Sn}}_{x}$Te and ${\text{Pb}}_{1-x}{\text{Sn}}_{x}$Se measured using optical techniques at different temperatures. By increasing the temperature (T), $x_{c}$ also increases. Data adapted from Dimmock et al.,^73^ Strauss,^74^ and Wu et al.^75^ (C) Schematic of how external factors, such as temperature, can affect $x_{c}$ by perturbing the $L_{6}^{+}$ and $L_{6}^{-}$ bands. When the energy separation between the $L_{6}^{+}$ and $L_{6}^{-}$ bands in the NI is large, $x_{c}$ is closer to the TI composition. (D) Room temperature weighted mobility of ${\text{Pb}}_{1-x}{\text{Sn}}_{x}$Te at different compositions, calculated using the reported conductivity and Seebeck coefficient.^50^ The blue shading is to guide the eye. Data adapted from Witting et al.,^39^ Orihashi et al.,^76^ Ortiz et al.,^77^ Kim et al.,^78^ Pang et al.,^79^ and Freer et al.^80^

Alloys between a normal insulator, which has non-inverted bands, and a TI, which has inverted bands, allow us to understand how TE properties evolve through a topological transition from one band structure topology to another. Through this transition, the band gap closes at some critical composition $x_{c}$, where the bands become linear Dirac cones (Figure 7A).

There are several factors that affect $x_{c}$, such as temperature.^73^^,^^74^^,^^81^ It is evident from optical measurements that increasing the temperature shifts $x_{c}$ toward a more Sn-rich composition in both ${\text{Pb}}_{1-x}{\text{Sn}}_{x}$Te and ${\text{Pb}}_{1-x}{\text{Sn}}_{x}$Se, for example, from $x_{c}\approx 0.4$ at 12 K to $x_{c}\approx 0.6$ at 300 K in ${\text{Pb}}_{1-x}{\text{Sn}}_{x}$Te (Figure 7B). Fundamentally, changes in the topological transition behavior can be explained by orbital chemistry. In IV–VI rock-salt alloys, the transition involves an inversion of the $L_{6}^{+}$ and $L_{6}^{-}$ bands (Figure 7A), which result from sp-mixing between the cation and anion (Figure 5A). Separating the cation and anion species by, e.g., increasing temperature would weaken sp-mixing, resulting in either a wider gap between the $L_{6}^{+}$ and $L_{6}^{-}$ bands in a normal insulator or a narrower gap in a TI (Figure 7C). In either case, $x_{c}$ would shift toward the TI-rich side, explaining the increase in $x_{c}$ with temperature in ${\text{Pb}}_{1-x}{\text{Sn}}_{x}$Te and ${\text{Pb}}_{1-x}{\text{Sn}}_{x}$Se (Figure 7B).

The band structure evolution through the topological phase transition should influence TE properties. If the majority carrier type remains the same across the transition, then one may expect a minimum in the carrier effective mass and a maximum in mobility near $x_{c}$. Indeed, the effective mass has been shown to reach a minimum near $x_{c}$ in ${\text{Pb}}_{1-x}{\text{Sn}}_{x}$Te.^82^^,^^83^ However, there have also been reports that appear to challenge expectations.^77^^,^^84^ Ortiz et al., for example, found that the room temperature intrinsic mobility of undoped ${\text{Pb}}_{1-x}{\text{Sn}}_{x}$Te reaches a maximum near x≈0.3^77^, as opposed to x≈0.6 where the topological transition is expected to occur (Figure 7B). Inconsistencies can arise because not all changes in properties are associated with changes in the band structure.

Interestingly, we find consistent agreement regarding one material property of undoped ${\text{Pb}}_{1-x}{\text{Sn}}_{x}$Te: the weighted mobility ($\mu_{w}$). By calculating $\mu_{w}$ from the measured electrical conductivity and Seebeck coefficient^50^ at room temperature across different studies,^76^^,^^77^^,^^78^^,^^79^ we find that it reaches a minimum near x≈0.6 (Figure 7D). Given that the alloy reaches a semimetallic state near this composition and semimetals typically exhibit low $\mu_{w}$ due to bipolar conduction effects,^85^ it is reasonable to say that the small $\mu_{w}$ is attributable to the topological transition in ${\text{Pb}}_{1-x}{\text{Sn}}_{x}$Te. Since the band structure evolution through the topological transition can be captured by a k·p band structure model,^86^ describing TE properties using an adapted transport model may help understand the origin of the weighted mobility trend.^87^

## Outlook

The research landscape is now more fertile than ever for exploring TIs for TE applications. Experimental and theoretical studies have jointly established a strong fundamental understanding of unconventional effects arising from TI surface states on TE properties. New discoveries of bulk transport phenomena in TIs, particularly those driven by band inversion, also create new opportunities for both fundamental and technology-oriented research that delve beneath the widely studied surface states. Bulk properties are especially important for practical applications such as Peltier coolers, which are typically on the scale of millimeters and operate near/above room temperature. There may also be yet-to-be-discovered TE phenomena in TIs that arise due to band inversion, perhaps those in transverse directions similar to the anomalous Nernst effect in the broader class of topological materials.^18^

At the moment, there is limited attention to bulk states and corresponding bulk charge transport properties in TIs. While there are many materials that have been studied for TE^10^^,^^88^ and TI^89^^,^^90^ applications separately, there are only a handful that have been studied for both. The immediate impact of studying bulk TE properties is the expansion of the “material genome” at the relatively unexplored intersection of TIs and TEs. Valuable insight can be gained from material-agnostic transport models,^43^ but concrete examples are needed to explore the complexities of real materials. Aside from the ones in this review, we believe that there are other known TI families where unconventional TE phenomena can be observed, such as ${\left(\text{SnSe}\right)}_{1-x}{\left(ABX_{2}\right)}_{x}$ alloys^91^^,^^92^^,^^93^^,^^94^^,^^95^^,^^96^^,^^97^ and the homologous series of ternary ${\left(\text{TrCh}\right)}_{n}{\left({\text{Pn}}_{2}{\text{Ch}}_{3}\right)}_{m}$ compounds, where Tr = tetrel (Ge, Sn, Pb), Pn = pnictogen (Sb, Bi), and Ch = chalcogen (Se, Te).^98^^,^^99^^,^^100^^,^^101^^,^^102^ A more holistic understanding of band inversion-driven effects, complete with generalized models and case studies of specific materials, will enrich design strategies to improve zT in TI-based TEs. Since commercial Peltier cooling devices are typically composed of TI alloys (${\text{Bi}}_{2}{\text{Te}}_{3-x}{\text{Se}}_{x}$ for the n-type leg and ${\text{Bi}}_{2-x}{\text{Sb}}_{x}{\text{Te}}_{3}$ for the p-type leg), a better understanding of bulk TE properties unique to TIs also holds technological and economical significance.

There are also avenues for discovering entirely new classes of high-performing TE materials within the space of TIs. Many TIs, in fact, have been suggested as candidates for TEs, particularly half-Heuslers^103^^,^^104^ and Zintl phases.^105^ Past recommendations often advertise the low κ_L_ stemming from the heavy atomic compositions of TIs, but exotic phenomena related to electronic transport, especially through the bulk, can also fuel future TE discovery ventures. With the advent of computational methods to rapidly predict TE properties^85^^,^^106^^,^^107^^,^^108^^,^^109^ combined with the availability of large-scale TI databases,^110^^,^^111^ it is an opportune time to explore new TEs within known and predicted TIs. Even entirely new material classes that are yet to be explored by either the TI or TE community can be investigated by coordinating high-throughput material discovery workflows. We anticipate that data-driven approaches will guide future TE research and reveal an entirely new frontier for TI research rooted in TE applications.

## Data and code availability

Data are available from the corresponding authors upon reasonable request.

## Acknowledgments

M.Y.T. is funded by the 10.13039/100000015US Department of Energy through the Computational Science Graduate Fellowship (DOE CSGF) under grant number DE-SC0020347. M.Y.T. also acknowledges support from the Johannes and Julia Randall Weertman Graduate Fellowship. The authors acknowledge the support of award 70NANB19H005 from the 10.13039/100000190US Department of Commerce, 10.13039/100000161National Institute of Standards and Technology, as part of the 10.13039/100014720Center for Hierarchical Materials Design (CHiMaD). The funders had no role in the study design, data collection and analysis, decision to publish, or preparation of the manuscript.

## Author contributions

Conceptualization, M.Y.T. and G.J.S.; investigation, M.Y.T.; data curation, M.Y.T.; formal analysis, M.Y.T.; writing – original draft, M.Y.T.; writing – editing, M.Y.T. and G.J.S.; supervision, G.J.S.; project administration, G.J.S. Both authors contributed to the manuscript and approved the final version.

## Declaration of interests

The authors declare no competing interests.
